# Long-term quality of life after endoscopic removal of sinonasal inverted papillomas: a 6-year cohort analysis in a tertiary academic hospital

**DOI:** 10.1007/s00405-015-3751-1

**Published:** 2015-08-18

**Authors:** Anusha van Samkar, Christos Georgalas

**Affiliations:** Department of ENT and Skull Base Center, Academic Medical Center, University of Amsterdam, Meibergdreef 9, 1105 AZ Amsterdam, The Netherlands; Department of Otorhinolaryngology, Academic Medical Center, University of Amsterdam, Amsterdam, The Netherlands

**Keywords:** SNOT-22, Sinonasal, Quality of life, Tumour, Inverted papilloma, Endoscopic

## Abstract

Inverted papillomas may affect the (para)nasal cavity. While some of these papillomas can undergo malignant transformation, others grow slowly and cause few if any symptoms. An endoscopic approach is seen as providing a balance between the greatest removal possible and avoiding unnecessary morbidity. However, the actual long-term quality of life of patients having undergone surgery for inverted papillomas has never been studied. Our primary aim is to assess the long-term sequelae and the quality of life of patients after endoscopic surgery of sinonasal inverted papillomas. The secondary aim is to establish which nasal symptoms, if any, are the most prevalent before and after surgery. We used the SNOT-22 questionnaire to assess the quality of life of patients who had undergone endoscopic surgery for sinonasal inverted papillomas between 2000 and 2011. Twenty-seven out of 34 patients returned the questionnaire (79 % response rate). Median follow-up was 6 years (range 1–10). Mean age was 58.9 years (range 40–85). Median SNOT-22 score was 12, while the most frequent postoperative symptom was the need to blow the nose (18 patients) and the most frequent preoperative symptom was nasal obstruction. Patients after endoscopic removal of sinonasal inverted papillomas return to an almost normal quality of life, as measured by the disease-specific questionnaire SNOT-22. The most frequent symptom was the need to blow the nose.

## Introduction

Inverted papillomas are benign tumours of the sinonasal cavity. They can cause local non-specific symptoms such as nasal obstruction, sneezing and rhinorrhoea, and potentially undergo malignant transformation [1]. For their removal an external approach was used in the past; however, endoscopic sinus surgery has been the gold standard for the last decades [2]. Currently, it is universally accepted that the vast majority of sinonasal benign lesions can be adequately managed by an endoscopic approach [3]. However, traditional external approaches still have a role and are occasionally used in combination with endoscopic surgery.

The operative morbidity of endoscopic surgery includes bleeding, orbital complications, cerebrospinal fluid leak and stenosis of the lacrimal pathway, with the complication rate ranging from 0 to 20 % [4, 5].

Most studies support the conclusion that endoscopic removal of inverted papilloma is associated with lower recurrence rates than the external approach [2, 3, 6].

However, unlike the extended approaches for skull base tumours, whose quality of life has been extensively studied [7, 8], we did not find any published studies regarding the quality of life of patients undergoing endoscopic surgery for the removal of sinonasal inverted papillomas.

### Aim of this study

Our primary aim is to assess the quality of life of patients after surgery of sinonasal inverted papillomas. We hypothesise that the radical removal of these tumours will result in a reduced quality of life for those patients compared to the healthy population. We expected the average score on the SNOT-22 questionnaire to be higher than 9.3 (the mean SNOT-22 score in the general population) [9].

## Patients and methods

### Participants

In this single-centre outcome study, patients between 18 and 90 years of age were included who had undergone surgery for sinonasal inverted papillomas from 2000 until 2011, in the Academic Medical Center (AMC) in Amsterdam, the Netherlands.

### Methods

To score the quality of life, the disease-specific questionnaire SNOT-22 was used and patients were told that participation was voluntary and that not returning the questionnaire would not affect the care they would receive.

### Statistical analysis

All statistical analyses were performed using IBM SPSS 22.0 statistical software (IBM Corp. Released 2013. IBM SPSS Statistics for Windows, Version 22.0. Armonk, NY: IBM Corp.c., Chicago, IL, USA). Descriptive statistics were calculated and reported for all measures. The Kolmogorov–Smirnov test and Shapiro–Wilk test were used to assess the distribution of the data. Median and range were used for the description of non-parametric data, while differences between groups were calculated with the Mann–Whitney *U* Test as appropriate.

## Results

Between the years 2000 and 2011, 37 patients were managed with sinonasal inverted papillomas. After excluding the patients who were treated by the external approach (2 patients) and the patients who were no longer alive (1 patient), there were 34 patients eligible for inclusion.

**Fig. 1:**
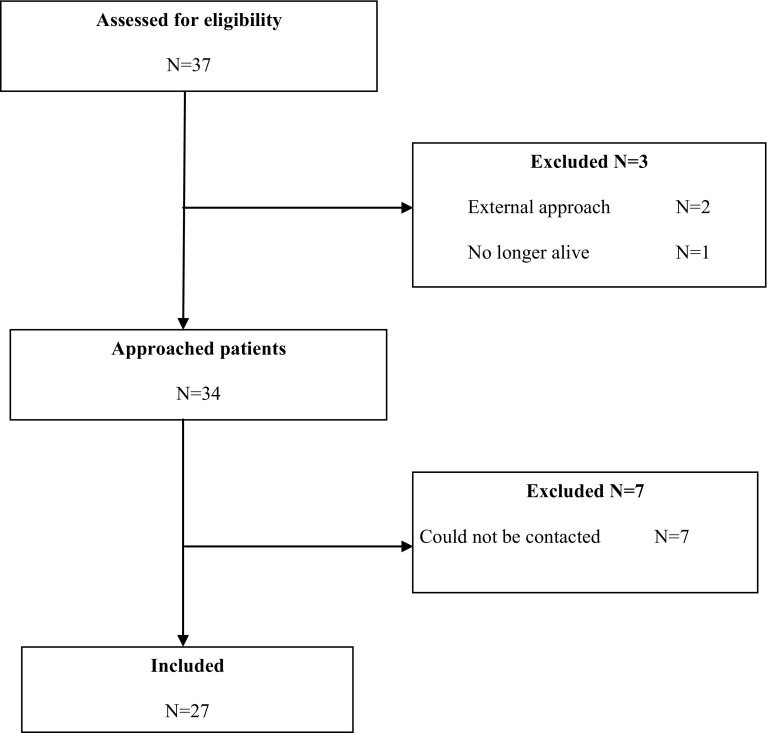
Flow-chart included patients

Of these, 27 patients returned the completed questionnaire (Fig. 1), resulting in a response rate of 27/34 = 79 %. Patients included 8 women and 19 men, with a mean age of 58.9 years (SD 9.8), and an age range of 40–85 years. Fifteen patients were under 60 years, 12 patients were 60 years or older. None of the patients had undergone radiation therapy in the past.

The median follow-up was 6 years, with a range of 1–10 years (Table 1).

**Table 1:** Follow-up and SNOT-22 score

Years of follow-up	Number of patients	Median SNOT-22 score (range)
1–5	12	11.5 (0–61)
6–10	15	12 (0–61)

*p* value: 0.683

The Kolmogorov–Smirnov test and Shapiro–Wilk test showed that distribution of the data was not normal (Kolmogorov–Smirnov: *p* = 0.000, Shapiro–Wilk: *p* = 0.000). Therefore, the Mann–Whitney *U* test was used for comparing groups, and medians were given instead of means for the SNOT-22 scores.

When comparing the medians of below and over 60 years of age, the median SNOT-22 score for the patients under 60 was 11.5, and for the patients of 60 and above it was 12 (Table 2). This was not significant (*p* = 0.456).

**Table 2:** Age and SNOT-22 score

Age	Number of patients	Median SNOT-22 score (range)
<60	15	13 (2–61)
≥60	12	11.5 (0–38)

*p* value: 0.456

The total median SNOT-22 score for all patients was 12.

The vast majority of patients had little or no nasal complaints. Scoring the SNOT 22, the most frequently reported symptom was the need to blow the nose; as reported by 18 out of 27 patients (median 1, range 0–5). Other symptoms reported were waking up at night (15 patients), postnasal and thick nasal drip (each 14 patients) and sneezing (13 patients). A median score per symptom is written in Table 3.

**Table 3:** Median scores per symptom and number of patients reporting the symptom

Symptom	Median (range)	Number of patients	Symptom	Median (range)	Number of patients
Need to blow the nose	1 (0–5)	18	Reduced sense of taste/smell	0 (0–5)	10
Waking up at night	1 (0–5)	15	Lack of good night sleep	0 (0–5)	9
Postnasal discharge	1 (0–4)	14	Cough	0 (0–3)	8
Thick nasal discharge	1 (0-5)	14	Dizziness	0 (0–5)	7
Sneezing	0 (0–5)	13	Reduced productivity	0 (0–3)	7
Reduced concentration	0 (0–4)	12	Frustration/restlessness/irritability	0 (0–4)	7
Blockage of the nose	0 (0–4)	12	Sadness	0 (0–4)	5
Waking up tired	1 (0–5)	11	Facial pain	0 (0–5)	4
Fatigue during the day	1 (0–5)	11	Embarrassment	0 (0–4)	4
Rhinorrhea	0 (0–4)	11	Earfullness	0 (0–2)	3
Difficulty falling asleep	0 (0–3)	10	Ear pain	0 (0–1)	2

Preoperative symptoms which led to consulting the Ear-, Nose-, Throat specialist were known in all patients. 19 patients (70 %) complained of nasal obstruction, 9 of rhinorrhoea and in three patients the inverted papilloma was found coincidentally when examining polyposis nasi. Other reported symptoms were, e.g., headache and a pressure feeling in head and/or face (Table 4). Twelve patients had multiple symptoms. There was no relationship between the type or number of preoperative symptoms and the postoperative SNOT-22 score.

**Table 4:** Reported reasons for consulting the ENT-specialist

Reported reason	Number of patients	Reported reason	Number of patients
Obstruction	19	Epistaxis	1
Rhinorrhoea	9	Felt an abnormality while picking nose	1
Headache	4	Sneezing	1
Pressure in head/face	4	Hyposmia	1
Occasionally at examination for polyposis nasi	3	Facial pain	1
Postnasal drip	2		

The extent of the surgery was known in all 27 patients (Table 5). Six underwent a wide local excision of the inverted papilloma, 16 underwent an ethmoidectomy and 5 underwent a medial maxillectomy. The nasolacrimal duct was cut in 2 patients; both underwent a medial maxillectomy and had a SNOT-22 score of 25. There was no significant difference in SNOT-22 scores between patients having undergone a wide local excision (median SNOT-22 score 18, range 5–38) and patients having undergone a medial maxillectomy (median SNOT-22 score 23, range 7–25, *p* = 0.792). SNOT-22 scores were lower in patients having undergone an ethmoidectomy (median SNOT-22 score 10.5, range 0–61), but the difference was not statistically significant (*p* = 0.203).

**Table 5:** Extent of endoscopic surgery performed

	Number of patients	Median SNOT-22 score (range)
Wide local excision	6	18 (5–38)
Ethmoidectomy	16	10.5 (0–61)
Medial maxillectomy	5	23 (7–25)
Including cut of the nasolacrimal duct	2	25 (25–25)

## Discussion

### Findings and analysis of the results

Our most important findings were that patients who had undergone surgery of benign sinonasal tumours appear to have a very mildly impaired quality of life (median of 12 on the SNOT-22 score). Healthy people score an average of 9.3 points on the SNOT-22 [9], while patients with chronic rhinosinusitis have an average of 51.8 [10]. The difference of 3 points is measurable but of doubtful clinical significance; it has been shown that a change of less than 9 points cannot be perceived as a real improvement or impairment by the patient [11].

A study that used SNOT-20 to assess the quality of life of patients after endoscopic removal of tumours was published by Harrow and co-authors [12]. Although the postoperative mean SNOT-20 score for benign tumours was 11.6 in their study, grossly equivalent to 13 in our scale, comparisons are difficult, as their study includes skull base tumours with significantly less follow-up and other kinds of benign sinonasal tumours which were not included in our study. In a way, our study is complementary to the study by Harrow, as it shows that quality of life continues to improve with time—our better reported quality of life may reflect the fact that their follow-up was 6 months, while ours was several years.

SNOT-22 questionnaires are also used for other sinonasal surgery. In all studies found, the SNOT-22 scores are higher than 9, which means there is an impaired quality of life after the operation; this confirms our findings. Buckland and co-authors report that the average postoperative SNOT-22 score in patients having undergone successful septal surgery is 19.3 after approximately 3 months [13]. Ransom and co-authors report an average SNOT-22 score of 14 a year after complete endoscopic resection of anterior skull base neoplasms [14]. The preoperative mean SNOT-22 score in their patient group was 47.

In our patient group, there is after 5 years a very minor, barely measurable and likely not of any clinical significance, quality of life impairment in patients who undergo an endoscopic excision of benign tumours.

The most frequent symptom reported in our patient group was the need to blow the nose, although the median score was reported as 1. This is likely reflective of a larger sinonasal cavity. Other frequent symptoms were waking up at night, postnasal discharge, thick nasal discharge and sneezing.

The most frequently reported preoperative symptoms were nasal obstruction and rhinorrhoea.

In studies about inverted papilloma and chronic rhinosinusitis, the most frequent symptom was nasal obstruction, which was confirmed in our study [4, 15, 16]. No postoperative patient group comparable to ours has been studied using the SNOT-22.

After analysis, it appeared we did not have to correct the results for the outliers. There are two patients scoring more than 60 on the SNOT-22 score, the result of unrelated co-morbidity (malignancy and depression). When these patients are removed from the database, the median becomes 11, suggesting that the results are not majorly influenced by those outliers.

Furthermore, the results did not have to be corrected for the extent of the endoscopic surgery, since there was no significant difference between the SNOT-22 scores in the different groups.

### Strengths and limitations

The strengths of our study include that the patients were approached personally and at different times of the day, to avoid just reaching the patients who were not working or not able to work. The long follow-up of the patients was a strength of the study, which is longer than current studies have investigated. One of the limitations of our research is that the patient group is small (27 patients) and that we could not reach 100 % of the patients. The majority of patients who did not respond appeared to have moved, which meant they could not be contacted by telephone or through mail. In a few, there may be nonresponse bias; patients without any symptoms, or, on the contrary, patients with major complaints and plenty of symptoms, may not have answered; however, an analysis of the non-responders did not show any obvious difference in age or preoperative symptoms. Furthermore, we do not have a preoperative SNOT-22 score to compare, as we are restricted by the retrospective nature of our study. However, we have reported preoperative symptoms, which give an indication for the reason of consulting the ENT-specialist.

## Conclusion

Our study shows that patients who have undergone surgery of benign sinonasal tumours appear to have a minor and probably not clinically significant impairment of quality of life (median of 12), as measured by the disease-specific questionnaire SNOT-22. This is present at a median of 6 years of follow-up. This may have implications for the consent process of such patients, who may have to be instructed accordingly.

The most frequent symptom was the need to blow the nose; 18 out of 27 patients reported this. Other frequent symptoms were waking up at night, postnasal and thick nasal discharge, and sneezing. The least frequent symptom was ear pain.

This was the first study assessing the quality of life of patients undergoing endoscopic removal of sinonasal inverted papillomas. Since only a small group of patients could be studied, it was not possible to draw firm conclusions. Further prospective and potentially multicentre studies may shed light on the quality of life of the patients before and after surgery.

## References

[1] Mirza S, Bradley PJ, Acharya A, Stacey M, Jones NS (2007). Sinonasal inverted papillomas: recurrence, and synchronous and metachronous malignancy. J Laryngol Otol.

[2] Busquets JM, Hwang PH (2006). Endoscopic resection of sinonasal inverted papilloma: a meta-analysis. Otolaryngol Head Neck Surg.

[3] Georgalas C, Fokkens WJ (2012) Rhinology and Skull Base Surgery: From the Lab to the Operating Room, chapter 43. The Role of Endoscopy in the Management of Benign and Malignant Sinonasal Tumours

[4] Durucu C, Baglam T, Karatas E, Mumbuc S, Kanlikama M (2009). Surgical treatment of inverted papilloma. J Craniofac Surg.

[5] Minovi A, Kollert M, Draf W, Bockmuhl U (2006). Inverted papilloma: feasibility of endonasal surgery and long-term results of 87 cases. Rhinology.

[6] Carta F, Verillaud B, Herman P (2011). Role of endoscopic approach in the management of inverted papilloma. Curr Opin Otolaryngol Head Neck Surg.

[7] Awad AJ, Mohyeldin A, El-Sayed IH, Aghi MK (2015). Sinonasal morbidity following endoscopic endonasal skull base surgery. Clin Neurol Neurosurg.

[8] Georgalas C, Badloe R, van Furth W, Reinartz S, Fokkens WJ (2012). Quality of life in extended endonasal approaches for skull base tumours. Rhinology.

[9] Gillett S, Hopkins C, Slack R, Browne JP (2009). A pilot study of the SNOT 22 score in adults with no sinonasal disease. Clin Otolaryngol.

[10] Sahlstrand-Johnson P, Ohlsson B, Von Buchwald C, Jannert M, Ahlner-Elmqvist M (2011). A multi-centre study on quality of life and absenteeism in patients with CRS referred for endoscopic surgery. Rhinology.

[11] Hopkins C, Gillett S, Slack R, Lund VJ, Browne JP (2009). Psychometric validity of the 22-item Sinonasal Outcome Test. Clin Otolaryngol.

[12] Harrow BR, Batra PS (2013). Sinonasal quality of life outcomes after minimally invasive resection of sinonasal and skull-base tumours. Int Forum Allergy Rhinol.

[13] Buckland JR, Thomas S, Harries PG (2003). Can the Sino-nasal Outcome Test (SNOT-22) be used as a reliable outcome measure for successful septal surgery?. Clin Otolaryngol Allied Sci.

[14] Ransom ER, Doghramji L, Palmer JN, Chiu AG (2012). Global and disease-specific health-related quality of life after complete endoscopic resection of anterior skull base neoplasms. Am J Rhinol Allergy.

[15] Bhattacharyya N (2006). Clinical and symptom criteria for the accurate diagnosis of chronic rhinosinusitis. Laryngoscope.

[16] Daines SM, Orlandi RR (2012). Chronic rhinosinusitis. Facial Plast Surg Clin North Am.

